# Supplementary figures and images for: Evaluating the effects of second-dose vaccine-delay policies in European countries: A simulation study based on data from Greece
Source: PLoS One. 2022 Apr 21;17(4):e0263977. doi: 10.1371/journal.pone.0263977 (PMC9022792; doi:10.1371/journal.pone.0263977)

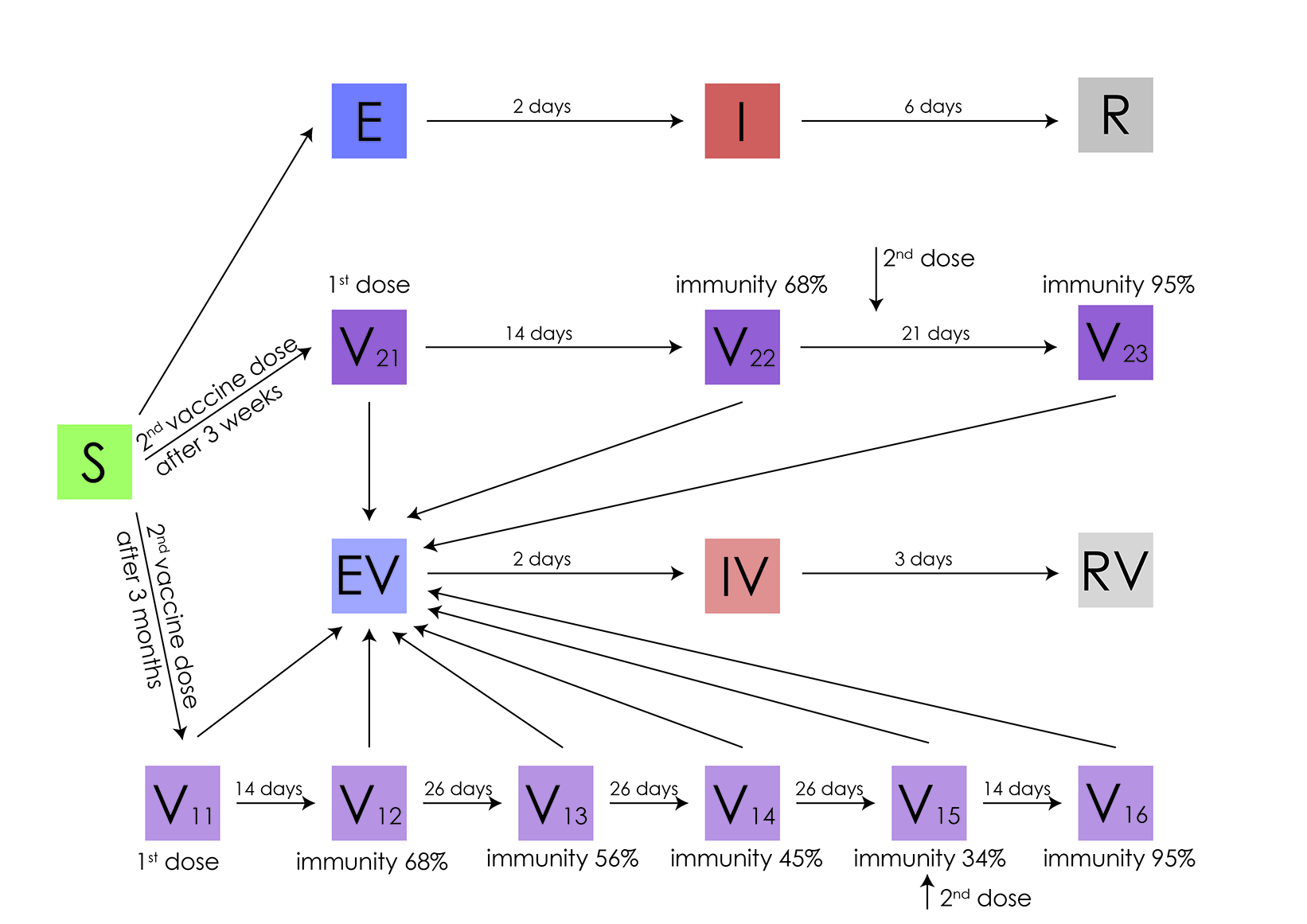

Supplement: S1 Fig — (TIF) [file pone.0263977.s001.tif]

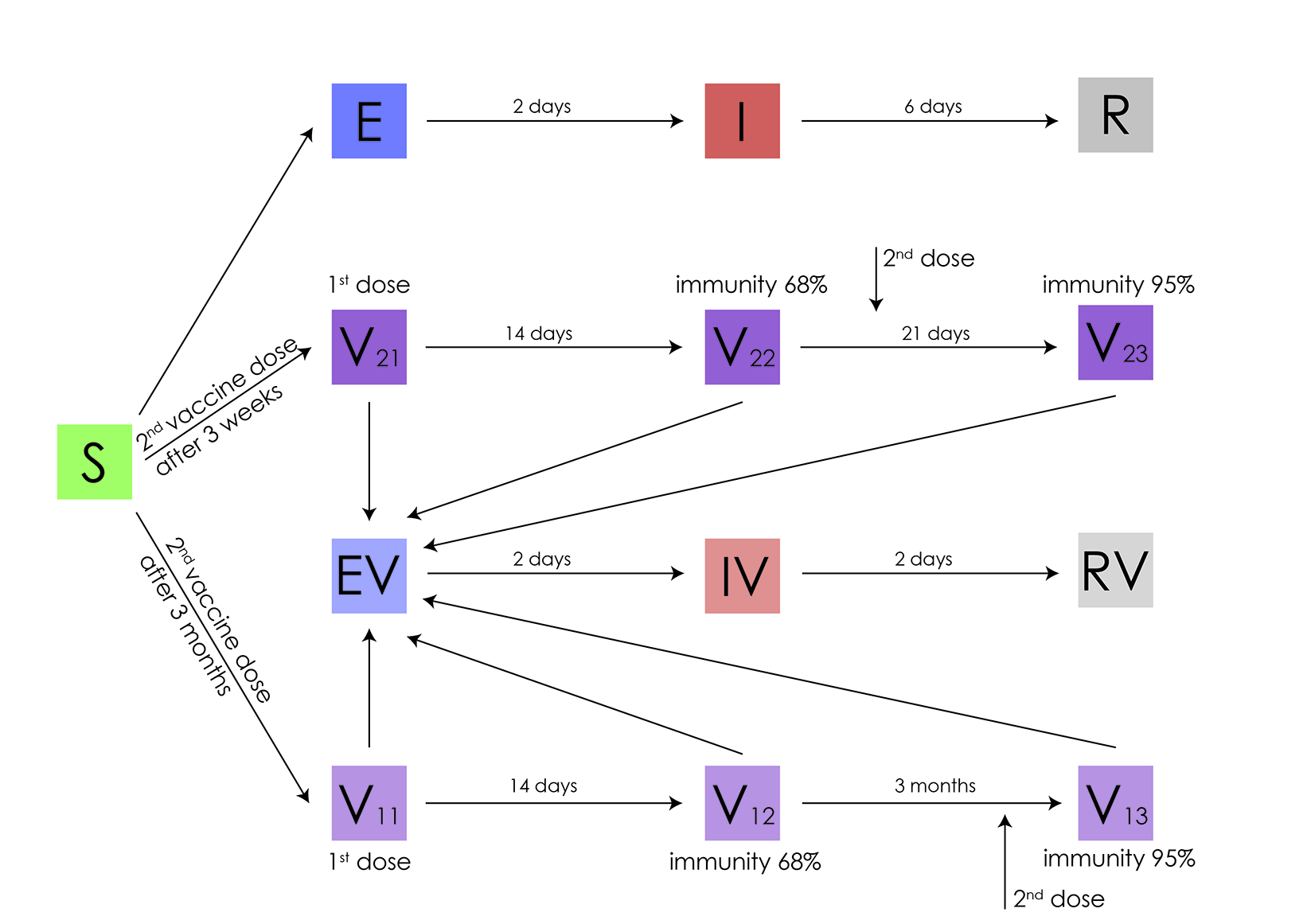

Supplement: S2 Fig — (TIF) [file pone.0263977.s002.tif]
