# Supplementary material for: Evaluating the effects of second-dose vaccine-delay policies in European countries: A simulation study based on data from Greece
Source: PLoS One. 2022 Apr 21;17(4):e0263977. doi: 10.1371/journal.pone.0263977 (PMC9022792; doi:10.1371/journal.pone.0263977)
Supplement: S1 Table — (DOCX) [file pone.0263977.s003.docx]

**S1 Table.** **Model Assumptions**

| **Parameters** | **Value** | **Comments-References** |
| --- | --- | --- |
| *R_t_* | 1.2  1.4 | Set to assess different levels of transmission.  *R_t_* is calculated as the largest eigenvalue of the next generation matrix, using an appropriate contact matrix (see below).  We consider reduced infection probability for children by 48% [39]. |
| Total population | 10816287 | Data from the Greek Statistics Authority [20]. |
| Population by age goup | 0-17: 1908003 (17.6%)  18-39: 3200713 (29.5%)  40-64: 3539972 (32.7%)  65+: 2167599 (20%) | Data from [20].  *S_initial_* = [*N*-R_initial_- I_initial_]  R_initial_ ~ 620000  I_initial_ ~60000. |
| Medical personnel population | Around 250000 | Rough estimate form data from [20] |
| Exposed period | 2 days for non-vaccinated and vaccinated people | Based on an average incubation time of approximately 5 days [26-28] and assuming that infectiousness starts approximately 2 days prior to the occurrence of symptoms [29-31]. |
| Duration of infectious period for non-vaccinated people | 6 days | Serial interval of approximately 6 days.  [32-34]. |
| Duration of infectious period for vaccinated persons | 3 days (worst case scenario)  2 days (baseline scenario and optimistic scenario, regarding vaccine efficiency) | Assuming, that vaccinations decrease the infectious period to one third (baseline scenario and optimistic scenario regarding vaccine efficiency) and to one half (worst case scenario). [35,36] |
| Age specific infection fatality ratios (IFR) | 0-17: 0.00003  18-39: 0.00020  40-64: 0.00500  65+: 0.05400 | Data from [37]. |
| Age specific infection fatality ratios (IFR) for vaccinated people | 0-17: 01.5e-06  18-39: 1.0e-05  40-64: 2.5e-04  65+: 2.7e-03 | We consider vaccinated people are 95% less probable of dying [38]. |
| Matrix of contacts between age groups  (0-17, 18-39, 40-64, 65+) | \|  \| *0-17* \| *18-39* \| *40-64* \| *65+* \| \| --- \| --- \| --- \| --- \| --- \| \| *0-17* \| 16.76 \| 4.34 \| 3.59 \| 0.46 \| \| *18-39* \| 2.55 \| 6.71 \| 3.47 \| 0.86 \| \| *40-64* \| 1.88 \| 3.10 \| 5.42 \| 0.98 \| \| *65+* \| 0.41 \| 1.32 \| 1.67 \| 1.41 \| | Based on social contacts surveys assessing contacts in Greece [41]; these data were collected in the second half of September 2020 . |
| **Parameters related to vaccine efficacy and roll-out** | | |
| Vaccine efficacy following the 1^st^ dose and before the 2^nd^ dose | 68% | 52.4% -92.6% [3-7]. [Assuming also reduction in the probability of acquiring infection]. This efficacy is reached 14 days post-vaccination. |
| Vaccine efficacy after the 2^nd^ dose | 95% | Vaccine efficacy in symptomatic infection [3-7]. [Assuming also reduction in the probability of acquiring infection.] This efficacy is reached 14 days post-vaccination. |
| Available doses over time – normal vaccine availability (25/1/2021) | We assumed that the number of doses increased during the first quarter of 2021 from approximately 81,000 to 2.5 million and that it ranged between 4.0 million to 6.3 million per quarter after March 2021. Estimates from government statements on media about vaccine roll out.   \| Period \| Doses \| \| --- \| --- \| \| 12/2020 \| 81000 \| \| 01/2021 \| 350000 \| \| 02/2021 \| 900000 \| \| 03/2021 \| 2500000 \| \| Q2 2021 \| 5800000 \| \| Q3 2021 \| 6300000 \| \| Q4 2021 \| 3700000 \| |  |
| Available doses over time – reduced Vaccine Availability (25/1/2021) | We assumed that the number of doses increased during the first quarter of 2021 from approximately 81,000 to 1 million and that it ranged between 4.0 million to 6.3 million per quarter after March 2021. Estimates from government statements on media about vaccine roll out.   \| Period \| Doses \| \| --- \| --- \| \| 12/2020 \| 81000 \| \| 01/2021 \| 35000 \| \| 02/2021 \| 90000 \| \| 03/2021 \| 100000 \| \| Q2 2021 \| 5800000 \| \| Q3 2021 \| 6300000 \| \| Q4 2021 \| 3700000 \| |  |
| Intention to get vaccinated (%) | \| *Age group* \| *Total* \| *Probably/Definitely Yes* \| *Probably/Definitely No* \| *Don’t know/ Don’t answer* \| \| --- \| --- \| --- \| --- \| --- \| \| *18-39* \| 329 \| 193 (58.7) \| 88 (26.8) \| 48 (14.6) \| \| *40-64* \| 418 \| 288 (68.9) \| 75 (17.9) \| 55 (13.2) \| \| *65+* \| 350 \| 277 (79.1) \| 36 (10.3) \| 37 (10.6) \| | Assessed in a sample of 1,097 adults [40] |
