# Supplementary material for: Evaluating the effects of second-dose vaccine-delay policies in European countries: A simulation study based on data from Greece
Source: PLoS One. 2022 Apr 21;17(4):e0263977. doi: 10.1371/journal.pone.0263977 (PMC9022792; doi:10.1371/journal.pone.0263977)
Supplement: S2 Table — (DOCX) [file pone.0263977.s004.docx]

**S2 table.** **Cumulative number of deaths, when 0% of vaccines allocated to ages 18-74, Baseline Scenario - Vaccine Availability - Rt=1.2**

| Cumulative Deaths | End of March | End of June | End of August | End of October | End of December |
| --- | --- | --- | --- | --- | --- |
| 0-17 | 6 (6-6) | 14 (14-14) | 15 (15-15) | 16 (16-16) | 17 (17-17) |
| 18-39 | 75 (74-75) | 143 (143-144) | 146 (145-146) | 146 (146-146) | 146 (146-146) |
| 40-64 | 1854 (1846-1863) | 3649 (3646-3652) | 3747 (3745-3749) | 3748 (3746-3750) | 3749 (3747-3751) |
| 65+ | 1897 (1886-1906) | 2384 (2373-2393) | 2425 (2416-2436) | 2461 (2451-2471) | 2495 (2485-2505) |
| Total deaths | 3832 (3812-3850) | 6190 (6176-6203) | 6333 (6321-6346) | 6371 (6359-6383) | 6407 (6395-6419) |
| Total life years lost | 73352.5 (72982-73685.5) | 134837.5 (134670.5-135044) | 138298.5 (138122-138435.5) | 138654 (138524-138784) | 138995.5 (138865.5-139125.5) |
