# Supplementary material for: Evaluating the effects of second-dose vaccine-delay policies in European countries: A simulation study based on data from Greece
Source: PLoS One. 2022 Apr 21;17(4):e0263977. doi: 10.1371/journal.pone.0263977 (PMC9022792; doi:10.1371/journal.pone.0263977)
Supplement: S3 Table — (DOCX) [file pone.0263977.s005.docx]

**S3 Table.** **Cumulative number of deaths, when 20% of vaccines allocated to ages 18-74, Baseline Scenario - Vaccine Availability - Rt=1.2**

| **Cumulative deaths** | End of March | End of June | End of August | End of October | End of December |
| --- | --- | --- | --- | --- | --- |
| 0-17 | 6 (6-6) | 14 (14-14) | 15 (15-15) | 16 (16-16) | 17 (17-17) |
| 18-39 | 75 (74-75) | 139 (139-139) | 141 (141-141) | 141 (141-141) | 141 (141-141) |
| 40-64 | 1852 (1844-1859) | 3550 (3547-3552) | 3632 (3630-3634) | 3634 (3631-3635) | 3635 (3632-3636) |
| 65+ | 1910 (1900-1919) | 2498 (2488-2506) | 2542 (2533-2551) | 2580 (2571-2589) | 2617 (2609-2626) |
| Total deaths | 3843 (3824-3859) | 6201 (6188-6211) | 6330 (6319-6341) | 6371 (6359-6381) | 6410 (6399-6420) |
| Total life years lost | 73383.5 (73020-73656.5) | 132451.5 (132291.5-132567.5) | 135400 (135277-135523) | 135799.5 (135646.5-135892.5) | 136162 (136016-136255) |
