# Supplementary material for: Evaluating the effects of second-dose vaccine-delay policies in European countries: A simulation study based on data from Greece
Source: PLoS One. 2022 Apr 21;17(4):e0263977. doi: 10.1371/journal.pone.0263977 (PMC9022792; doi:10.1371/journal.pone.0263977)
Supplement: S4 Table — (DOCX) [file pone.0263977.s006.docx]

**S4 Table.** **Cumulative number of deaths, when 50% of vaccines allocated to ages 18-74, Baseline Scenario - Vaccine Availability - Rt=1.2**

| **Cumulative deaths** | End of March | End of June | End of August | End of October | End of December |
| --- | --- | --- | --- | --- | --- |
| 0-17 | 6 (6-6) | 13 (13-13) | 15 (15-15) | 16 (16-16) | 17 (17-17) |
| 18-39 | 74 (74-75) | 131 (131-132) | 132 (132-132) | 132 (132-132) | 132 (132-132) |
| 40-64 | 1847 (1838-1853) | 3366 (3363-3369) | 3419 (3417-3422) | 3422 (3419-3424) | 3423 (3420-3425) |
| 65+ | 1928 (1919-1941) | 2723 (2713-2735) | 2772 (2762-2784) | 2816 (2806-2827) | 2858 (2848-2869) |
| Total deaths | 3855 (3837-3875) | 6233 (6220-6249) | 6338 (6326-6353) | 6386 (6373-6399) | 6430 (6417-6443) |
| Total life years lost | 73306 (72973-73630.5) | 128005 (127845-128232.5) | 130138.5 (130008.5-130312.5) | 130610 (130450-130747) | 131007.5 (130847.5-131144.5) |
