# Supplementary material for: Evaluating the effects of second-dose vaccine-delay policies in European countries: A simulation study based on data from Greece
Source: PLoS One. 2022 Apr 21;17(4):e0263977. doi: 10.1371/journal.pone.0263977 (PMC9022792; doi:10.1371/journal.pone.0263977)
Supplement: S5 Table — (DOCX) [file pone.0263977.s007.docx]

**S5 Table. Cumulative number of deaths, when 100% of vaccines allocated to ages 18-74, Baseline Scenario - Vaccine Availability - Rt=1.2**

| **Cumulative deaths** | End of March | End of June | End of August | End of October | End of December |
| --- | --- | --- | --- | --- | --- |
| 0-17 | 6 (6-6) | 13 (13-13) | 15 (15-15) | 16 (16-16) | 17 (17-17) |
| 18-39 | 75 (74-75) | 129 (129-130) | 130 (130-130) | 130 (130-130) | 130 (130-130) |
| 40-64 | 1854 (1846-1862) | 3304 (3300-3309) | 3311 (3307-3316) | 3313 (3310-3319) | 3314 (3311-3319) |
| 65+ | 1881 (1870-1889) | 2278 (2268-2286) | 2312 (2302-2320) | 2340 (2330-2348) | 2367 (2357-2375) |
| Total deaths | 3816 (3796-3832) | 5724 (5710-5738) | 5768 (5754-5781) | 5799 (5786-5813) | 5828 (5815-5841) |
| Total life years lost | 73240.5 (72870-73536.5) | 122923 (122733-123182.5) | 123571.5 (123381.5-123777.5) | 123901 (123741-124137) | 124193.5 (124033.5-124399.5) |
