# Supplementary material for: Evaluating the effects of second-dose vaccine-delay policies in European countries: A simulation study based on data from Greece
Source: PLoS One. 2022 Apr 21;17(4):e0263977. doi: 10.1371/journal.pone.0263977 (PMC9022792; doi:10.1371/journal.pone.0263977)
Supplement: S6 Table — (DOCX) [file pone.0263977.s008.docx]

**S6 Table.** **Cumulative number of infections, when 0% of vaccines allocated to ages 18-74, Baseline Scenario - Vaccine Availability - Rt=1.2**

| **Cumulative infections** | End of March | End of June | End of August | End of October | End of December |
| --- | --- | --- | --- | --- | --- |
| 0-17 | 205692 (201056-210308) | 407403 (395412-419507) | 449126 (434390-464064) | 483022 (465868-500426) | 514726 (495223-534530) |
| 18-39 | 355723 (349647-361850) | 657034 (642333-671954) | 675324 (658976-691931) | 678502 (661464-695861) | 681604 (663874-699700) |
| 40-64 | 354386 (348331-360467) | 668880 (654066-683859) | 695936 (679082-713033) | 699982 (682338-717904) | 703266 (684908-721941) |
| 65+ | 42180 (40320-44094) | 57708 (54237-61357) | 59220 (55278-63394) | 60525 (56142-65195) | 61809 (56997-66971) |
