# Supplementary material for: Evaluating the effects of second-dose vaccine-delay policies in European countries: A simulation study based on data from Greece
Source: PLoS One. 2022 Apr 21;17(4):e0263977. doi: 10.1371/journal.pone.0263977 (PMC9022792; doi:10.1371/journal.pone.0263977)
Supplement: S7 Table — (DOCX) [file pone.0263977.s009.docx]

**S7 Table.** **Cumulative number of infections, when 20% of vaccines allocated to ages 18-74, Baseline Scenario - Vaccine Availability - Rt=1.2**

| **Cumulative infections** | End of March | End of June | End of August | End of October | End of December |
| --- | --- | --- | --- | --- | --- |
| 0-17 | 205563 (201024-210191) | 403485 (391605-415485) | 445046 (430414-459859) | 479322 (462237-496643) | 511262 (491857-531012) |
| 18-39 | 355280 (349268-361332) | 643554 (628986-658220) | 662802 (646528-679252) | 668244 (651052-685670) | 671562 (653659-689750) |
| 40-64 | 353940 (347968-360029) | 655699 (641042-670620) | 682914 (666185-699958) | 689238 (671522-707328) | 693104 (674617-712012) |
| 65+ | 42170 (40289-44097) | 58765 (55217-62461) | 60310 (56284-64541) | 61671 (57194-66410) | 63013 (58098-68248) |
