# Supplementary material for: Evaluating the effects of second-dose vaccine-delay policies in European countries: A simulation study based on data from Greece
Source: PLoS One. 2022 Apr 21;17(4):e0263977. doi: 10.1371/journal.pone.0263977 (PMC9022792; doi:10.1371/journal.pone.0263977)
Supplement: S8 Table — (DOCX) [file pone.0263977.s010.docx]

**S8 Table.** **Cumulative number of infections, when 50% of vaccines allocated to ages 18-74, Baseline Scenario - Vaccine Availability - Rt=1.2**

| **Cumulative infections** | End of March | End of June | End of August | End of October | End of December |
| --- | --- | --- | --- | --- | --- |
| 0-17 | 205382 (200846-210114) | 396362 (384695-408329) | 437560 (423185-452361) | 472510 (455670-489830) | 504882 (485694-524631) |
| 18-39 | 354592 (348511-360648) | 618118 (603982-632366) | 638982 (623014-655121) | 646856 (629797-664156) | 650152 (632378-668213) |
| 40-64 | 353262 (347216-359440) | 630735 (616477-645284) | 658194 (641843-674894) | 667522 (649974-685486) | 671722 (653378-690546) |
| 65+ | 42128 (40257-44041) | 60744 (57067-64557) | 62360 (58188-66723) | 63826 (59184-68701) | 65248 (60157-70642) |
