# Supplementary material for: Evaluating the effects of second-dose vaccine-delay policies in European countries: A simulation study based on data from Greece
Source: PLoS One. 2022 Apr 21;17(4):e0263977. doi: 10.1371/journal.pone.0263977 (PMC9022792; doi:10.1371/journal.pone.0263977)
Supplement: S9 Table — (DOCX) [file pone.0263977.s011.docx]

**S9 Table.** **Cumulative number of infections, when 100% of vaccines allocated to ages 18-74, Baseline Scenario - Vaccine Availability - Rt=1.2**

| **Cumulative infections** | End of March | End of June | End of August | End of October | End of December |
| --- | --- | --- | --- | --- | --- |
| 0-17 | 205698 (201113-210283) | 394808 (383203-406569) | 434849 (420571-449360) | 470048 (453294-487112) | 502502 (483371-521985) |
| 18-39 | 355640 (349658-361635) | 611915 (598340-625688) | 638422 (622750-654357) | 645310 (628653-662313) | 648467 (631115-666220) |
| 40-64 | 354362 (348317-360332) | 623402 (609535-637325) | 651674 (635668-667804) | 661004 (643853-678331) | 664376 (646514-682482) |
| 65+ | 42111 (40235-44022) | 59390 (55683-63234) | 61865 (57562-66355) | 63053 (58343-68022) | 64212 (59091-69658) |
